# Comparison of the epidemiological aspects of acute infectious diseases between foreign and native imported cases in the border counties of Southwest China, 2008–2017

**DOI:** 10.1017/S0950268819001195

**Published:** 2019-06-28

**Authors:** Li Jiang, Tian Huang

**Affiliations:** 1 The First Affiliated Hospital of Guizhou University of Traditional Chinese Medicine, Guiyang, Guizhou, China; 2 Yunnan Provincial Center for Disease Control and Prevention, Kunming, Yunnan, P.R, China

**Keywords:** Cross-border transmission, epidemiology, surveillance

## Abstract

This study analysed the epidemiological characteristics of imported cases of acute infectious diseases and compared these features by nationality in 25 border counties of Yunnan Province from 2008 to 2017 to inform prevention strategies. Surveillance data for the imported cases collected in the border counties were analysed to determine disease variety, seasonal patterns, infection site and personal demographics and these features were compared by patient nationality. A total of 12 820 imported cases were reported in the 25 border counties, with 5610 foreign cases and 7210 native cases. The disease spectrum was more diverse among foreign cases than among native cases. Both foreign and native cases were mostly imported from Myanmar. The shift in the number of foreign cases was greater than that of native cases after 2016. Ruili, a city, that shares a border with Myanmar, exhibited the greatest number of imported infections. Farmers, businessmen, women and preschool children were frequently diagnosed with infections. Multiple prevention strategies including disease screening at the border, health education before departure and health service provision to foreigners should be carried out to reduce the risk of autochthonous spreading and to avoid potential outbreaks. Furthermore, international collaboration in terms of sharing infectious disease data should be improved between China and neighbouring countries.

## Background

Globalisation processes are progressively increasing the flow of people across political and geographic boundaries. Data from the China National Tourism Administration show that 122 million people exited China and 138 million people entered China from foreign countries in 2016 [1]. This large population migration is increasing the potential risk of cross-border spreading of infectious diseases [2]. From 2013 through 2016, a total of 16 206 imported infectious disease cases were reported in China. Yunnan Province reported the largest number of cases [3].

**Fig. 1.**
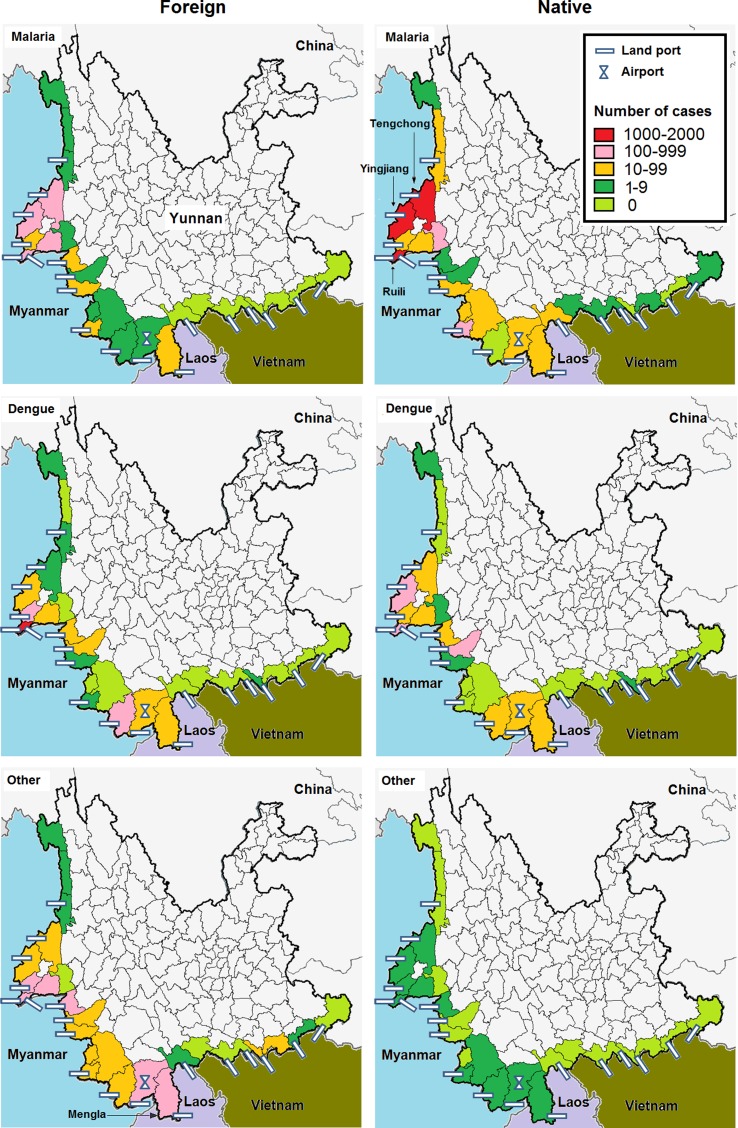
Number of malaria, dengue and others infectious diseases between foreign and native imported cases in the border counties of Southwest China, 2008–2017.

Yunnan is the southwestern-most province in China and shares a border of 4060 km with Myanmar in the west, Laos in the south and Vietnam in the southeast (Fig. 1). Twenty-five of 129 counties in the province are located on the border. These border counties have 19 border land ports, one international airport and over 90 routes that lead to neighbouring countries. Additionally, the population of these counties is more than 6.9 million, of which nearly 60% are ethnic minorities [4]. Due to the environmental continuity and closed cultural, historical and linguistic ties, cross-border marriage and trade are common [5]. The number of border entries and exits reached 40.6 million in 2017 [6]. This large cross-border population movement creates great challenges for preventing the transmission of infectious diseases in the border counties.

Disease surveillance plays an import role in the discovery of this epidemic situation and in responses for prevention and control [7]. A recent study carried out active surveillance at international entry-exit air, water and land ports in China from 2014 to 2016 [8]. The number of infectious diagnoses at land entry-exit stations contributed to the highest proportion of cases with mucocutaneous infection and the lowest proportion of cases with vector-borne infection. Furthermore, more than half of patients diagnosed in Yunnan had blood-transmitted and sexually transmitted infections, which was slightly higher than the proportion of vector-borne infections in the province. On the other hand, passive surveillance data based on the National Notifiable Infectious Diseases Reporting System (NNIDRS) were analysed from 2013 through 2016 [3]. Vector-borne infections had the highest proportion among imported cases. Most of these cases were reported in Yunnan. Although, both of these studies indicated that Yunnan suffered a great threat from the cross-border transmission of infectious diseases, the details of the epidemiological features were not explored, particularly in the border counties.

In recent years, with the high speed of China's economic growth, the border counties have received a large amount of workers and schoolchildren from neighbouring countries and have exported many tourists and businessmen to these counties [9–11]. Both the incoming people from the neighbouring countries and the returning Chinese nationals have been exposed to the different natural and social environments outside China. These people may be infected and carry diseases into China. However, due to the differences in living habits, the chance of exposure and vaccination histories, these factors may cause a different spectrum of infection between these foreign citizens and Chinese nationals. Thus, we analysed surveillance data from the NNIDRS to determine the epidemiological characteristics of acute infectious diseases among imported cases by nationality in the 25 border counties in Yunnan. These results will provide information for infectious disease cross-border prevention and control efforts.

## Method

### Surveillance

According to the *Law of the People's Republic of China on the Prevention and Control of Infectious Diseases*, 39 notifiable infectious diseases are classified into categories A, B and C depending on the mode of transmission, the speed of spread and the severity of disease. Class A, the most severe among the three classes, includes the plague and cholera, which are required to be reported within 2 h after diagnosis. Class B includes 26 diseases such as viral hepatitis and typhoid. Class C includes 11 diseases, such as influenza and mumps. Although infections from both classes B and C are required to be reported within 24 h after diagnosis, patients with diseases in class B should be isolated more strictly than patients with diseases in class C.

Cases of notifiable infectious diseases are reported via NNIDRS. This system is a passive, countrywide, web-based, real-time network that was established by the Chinese Center for Disease Control and Prevention (CDC) in 2004. The network extends to all hospitals and health centres, where data for cases, including demographic characteristics, diagnosis, onset and diagnosis data and epidemiological data (such as entry-exit history), are collected and entered into an electronic form by hospital or health care centre epidemiologists. The data are immediately sent to the national database where Chinese CDC officers at different levels can access the information within their jurisdiction, as soon as it is available.

### Case definition

Twenty-three reportable infectious diseases, including malaria, dengue fever, Japanese encephalitis, scrub typhus, hand-foot-and-mouth disease, viral hepatitis (A or E), shigellosis, other infectious diarrhoeas, typhoid and paratyphoid, cholera, influenza, measles, mumps, rubella, scarlatina, pertussis, epidemic cerebrospinal meningitis, gonorrhoea, acute haemorrhagic conjunctivitis, rabies, tetanus, leptospirosis and cutaneous anthrax, were investigated in this study. These diseases are characterised by symptoms of acute onset with a clear epidemiological exposure history and are diagnosed by local hospital or health centre clinicians. The latent period of each infection is listed in Supplementary Table S1.

Viral hepatitis (B, C or D), HIV/AIDS, syphilis and tuberculosis were excluded from this study. Because these diseases have a relatively long latent period and are endemic in the border counties, multiple infection sources exist. Thus, the location of acquisition is difficult to establish. Plague, leishmaniasis, schistosomiasis, filariasis, echinococcosis, leprosy, SARS, poliomyelitis, human infection with highly pathogenic avian influenza, amoebic dysentery, brucellosis, diphtheria and avian influenza H7N9 subtype infection were not detected by the surveillance system during the study period.

An imported case was defined as an infected person who was diagnosed with one of 23 acute infectious diseases in the 25 border counties and could be traced back to an exposure origin in Myanmar, Laos, or Vietnam within a latent period. According to nationality, an imported case was classified into either the foreign or native group.

### Data collection and statistics

Imported cases that were recorded in the Chinese NNIDRS database from 2008 through 2017 were downloaded and transferred into the R program (version 3.2.1) for data exploration and analysis. The infectious diseases were divided into vector-borne, gastrointestinal, respiratory and mucocutaneous infections by their transmission route. The variety of the infectious diseases was illustrated by the proportions among cases and origin countries. The seasonal pattern was described by year and month. The case locations were marked by the county to explore their geographic distribution in MapInfo (version 15, serial number: MINWCA 1500000240). Demographic characteristics, including age, gender, occupation, country of origin and period from onset of symptoms to diagnosis by physicians, were presented as percentages and medians, and the significance of the differences between foreign and native cases was initially assessed with the chi-square test or rank sum test. Temporal shifts in sex, age and occupation among cases in the two groups were presented by year and disease.

## Results

### Variety of diseases

During 2008–2017, 12 820 imported cases of acute infectious disease were reported in the 25 border counties of Yunnan, with 5610 foreign cases and 7210 native cases. Both foreign and native cases were mostly of Myanmar origin.

**Fig. 2.**
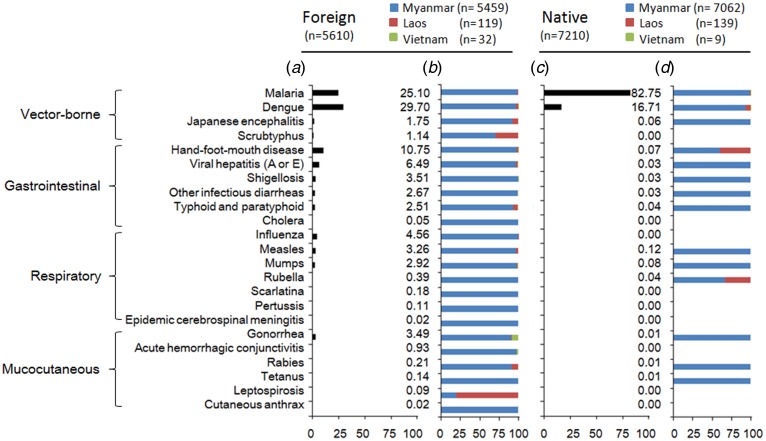
Proportion of each infection (a) and countries origin (b) among foreign imported cases and among native imported cases (c, d) in the border counties of Southwest China, 2008–2017.

All 23 types of the acute infectious diseases were found among the foreign cases. Vector-borne infectious disease comprised 58% (3236/5610) of the cases. The most frequently reported vector-borne pathogen was dengue, followed by malaria. Hand-foot-and-mouth disease, influenza and gonorrhoea were the most common among the gastrointestinal, respiratory and mucocutaneous infections, respectively (Fig. 2a). Infections of Myanmar origin contributed more than 75% of each infectious disease, except for scrub typhus and leptospirosis (Fig. 2b).

Only 14 types of the acute infectious diseases were reported among native cases. More than 99% (7175/7210) of the cases involved vector-borne infections, mostly malaria or dengue (Fig. 2c). Myanmar was a major origin of these infectious diseases (Fig. 2d).

### Seasonal patterns

The number of native cases was higher than that of foreign cases from 2008 to 2011. Then, the number of cases in the two groups trended towards similar numbers from 2012 to 2015. In contrast, foreign cases exceeded native cases in 2016 and 2017.

Malaria infections comprised the majority of total foreign cases, with epidemic peaks in May or June in 2008 and 2011. Dengue infections were occasionally observed during this period. However, after 2013, dengue infections markedly increased and peaked at 394 cases in October 2017. Other infections were detected sporadically during each month of the study period.

**Fig. 3.**
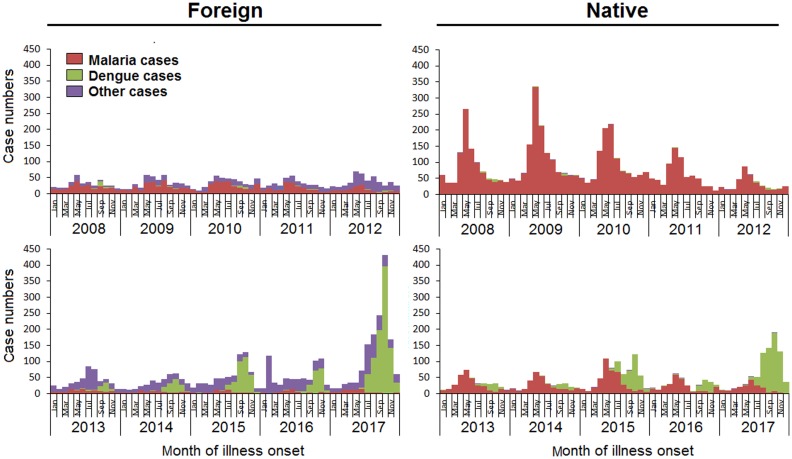
Yearly and monthly variation between foreign and native imported cases in the border counties of Southwest China, 2008–2017.

Seasonal patterns similar to those of foreign cases were observed among native cases. Malaria or dengue infections had peaks in May or October, respectively, each year. Malaria infections decreased each year, while dengue infections increased steadily (Fig. 3)

### Geographic distribution

The counties bordering Vietnam and northern Myanmar had a low incidence of both native and foreign cases. Malaria cases were mostly reported from the Ruili area, which shares a border with Myanmar. The incidence of native cases was higher than that of foreign cases in the area, particularly in the Ruili, Yingjiang and Tenchong counties. However, the incidence of dengue and other infectious diseases was higher among foreign cases than among native cases in most counties. High incidence counties were most frequently observed in the Ruili and Mengla areas, which share borders with Myanmar and Laos (Fig. 1).

### Characteristics of subjects

Males accounted for most of the cases, as indicated by male-to-female ratios of 1.53:1 and 5.36:1 among foreign and native cases, respectively. The median age of the foreign cases was 21 years old. Farmers, preschool children and students were the leading three occupation groups. However, the median age of the native cases was 34 years old. Farmers accounted for the highest proportion in this group, followed by labourers and businessmen. The median period from onset of symptoms to diagnosis by physicians was 3 days in both groups. Disease type and location of origin presented more diversity among foreign cases than among native cases (Table 1).

**Table 1.** Demographic characteristics between foreign and native imported cases in the border counties of Southwest China, 2008–2017

Items	Foreign *n* (%)	Native *n* (%)	χ^2^ test *P* value
Gender			<0.001
Female	2218 (39.54)	1133 (15.71)	
Male	3392 (60.46)	6077 (84.29)	
Age (years)			<0.001
<7	1293 (23.05)	121 (1.68)	
7–17	969 (17.27)	269 (3.73)	
18–64	3209 (57.20)	6739 (93.47)	
>64	139 (2.48)	81 (1.12)	
Median (IQR)	21 (7.25,32)	34 (2743)	<0.001^a^
Occupation			<0.001
Famer	1952 (34.8)	3486 (48.35)	
Pre-school child	1403 (25.01)	133 (1.84)	
Student	669 (11.93)	165 (2.29)	
Businessman	478 (8.52)	567 (7.86)	
Labour	476 (8.48)	2568 (35.62)	
Others	632 (11.27)	291 (4.04)	
Period			0.040^a^
Median (IQR)	3 (1,5)	3 (1,5)	
Infectious disease by transmission route			<0.001
Vector-borne	3236 (57.68)	7175 (99.51)	
Gastrointestinal	1458 (25.99)	14 (0.19)	
Respiratory	642 (11.44)	18 (0.25)	
Mucocutaneous	274 (4.88)	3 (0.04)	
Country origin			<0.001
Myanmar	5459 (97.31)	7062 (97.95)	
Laos	119 (2.12)	139 (1.93)	
Vietnam	32 (0.57)	9 (0.12)	

IQR, interquartile range.

a Rank sum test; period from onset of symptoms to diagnosis by physicians.

### Temporal shift of demographic profiles

**Fig. 4.**
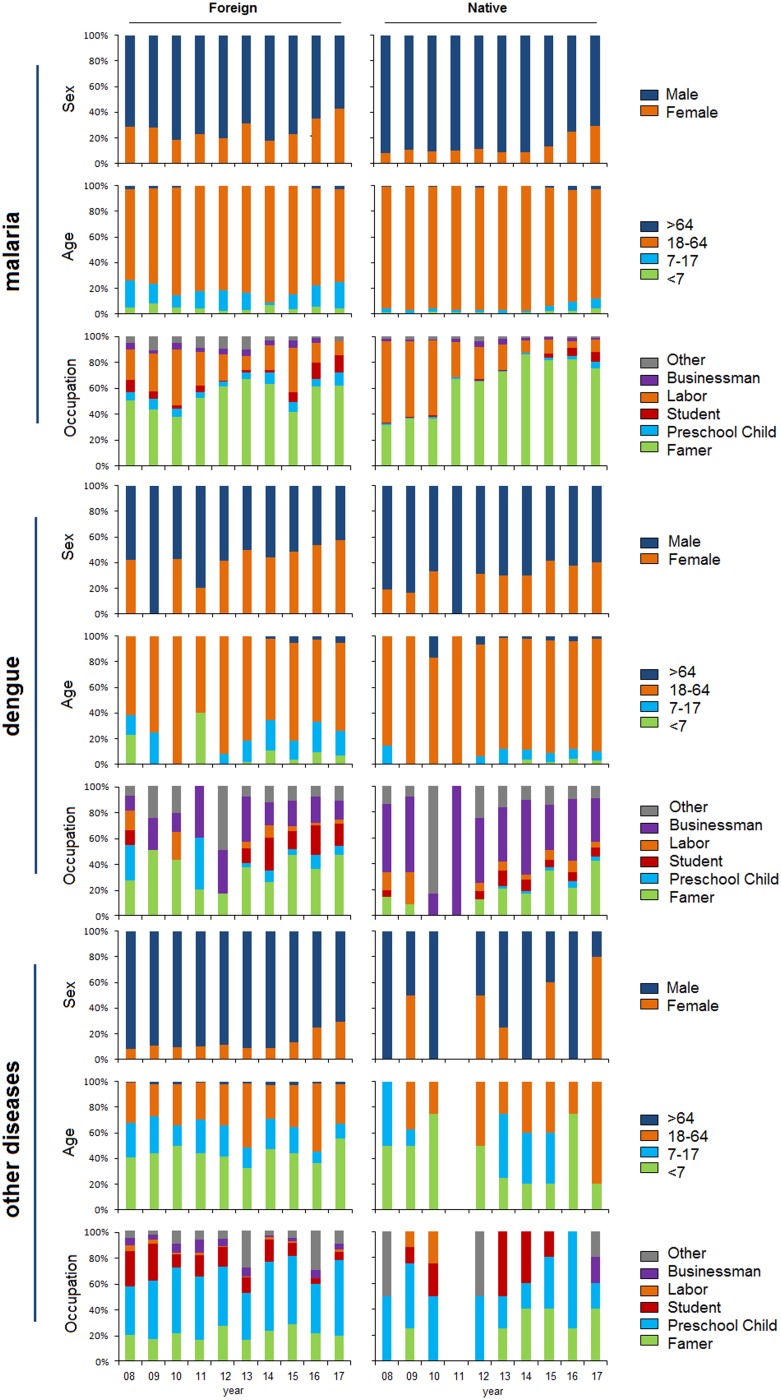
Sex, age and occupation by year and disease among foreign and native imported cases in the border counties of Southwest China, 2008–2017.

Female cases gradually increased each year in both the native and foreign groups. The number of foreign female cases exceeded that of male cases with dengue infection in 2016 and 2017. An age of 18–64 years old was predominant in the two groups with malaria or dengue infections. However, ages under 18 years old were mostly found with other infections. Farmers accounted for a high proportion of cases by occupation. Cases in laborers sharply decreased in both groups, particularly among native cases with malaria infection. Additionally, farmers, businessmen and preschool children were most frequently diagnosed with malaria, dengue and other infectious diseases, respectively (Fig. 4).

## Discussion

During our study period, 12 820 imported cases of acute infectious diseases, with 5610 foreign and 7210 native cases, were detected in the 25 border counties. Malaria was the predominant infection, particularly among native cases. Migrant labourers returning from Myanmar were considered to be important contributors [12]. Chinese companies working on development projects in agriculture and industry, such as the Myitsone Dam project, have typically brought their own Chinese workforce to meet their labour demands [13]. The labourers worked and lived in Myanmar, where malaria was highly endemic and prevention measures were lacking, which caused a high risk of infection. Since 2010, the number of Chinese labourers in Myanmar has decreased [12]. The number of imported malaria cases also fell markedly among native cases. On the other hand, members of the Greater Mekong Subregion (GMS) (composed of Cambodia, Yunnan and Guangxi provinces in China, Laos, Myanmar, Thailand and Vietnam) have accelerated their efforts to prevent, diagnose and treat malaria in recent years. The reported number of malaria cases and deaths in the GMS fell by 74% and 91%, respectively, between 2012 and 2016 [14]. These achievements also contributed to the decrease in imported malaria cases. Since 2017, China has announced the elimination of indigenous malaria [15]. Thus, it is necessary to prevent the re-emergenceof malaria by isolating and treating imported cases in border areas.

While malaria cases have been successfully reduced, dengue infection increased rapidly and exhibited a similar rising pattern among both foreign and native cases. Globally, the rapid geographical expansion of the virus has caused more than 390 million infections, with 96 million clinically symptomatic cases annually in more than 100 endemic countries [16]. Unlike malaria cases, which were composed mainly of labourers, businessmen accounted for a large proportion of native dengue cases in our study. Although both malaria and dengue are mosquito-borne diseases, there are different transmission patterns for the two pathogens. Malaria is mostly a rural problem, spreading radially to urban areas through the movement of people. Dengue, on the other hand, spreads from the larger cities (where it is often linked to unplanned urbanisation, standing water and weak sanitation systems) to smaller communities [17]. Businessmen were found as a risk group due to their frequent visits to areas with a high population density. However, as long as dengue vaccines and antiviral drugs remain a future prospect, prevention will continue to rely on vector control. Education programmes focused on protection against mosquito bites among tourists would be helpful for reducing the probability of dengue infection among Chinese nationals who frequently take cross-border trips. In addition, fever screening was shown to be useful in identifying 45% of 542 known imported dengue cases with fever at Taiwan airports [18]. Thus, this tool should be enhanced at border ports to reduce the number of foreign cases imported from the neighbouring countries.

Other infectious diseases were frequently observed among foreign cases and were mostly of Myanmar origin. Similar to the economic and political diversity of the three neighbouring countries, the incidence of communicable diseases and healthcare services also varies [7]. Only 3% of the total government budget of Myanmar is allocated to healthcare and allocations among regions and states are not proportional to health needs. Civil conflicts and the remoteness of some regions also contribute to poor coverage [19]. The lack of a good disease surveillance system and the inadequacy of the primary care infrastructure compound problems and make prevention, control and treatment of infectious diseases an urgent challenge. A region international collaboration, the Mekong Basin Disease Surveillance (MBDS) network, was formally established in 2001 [7]. Within the network, sharing surveillance data, responding to outbreaks and training public health experts would more effectively combat the disease threat in the region.

Hot spots for imported cases were located in the cross-border economic zone. The entry-exit population reached 17 million and 1 million at the Ruili (Yunnan)-Muse (Myanmar) and Mohan (Yunnan)-Boten (Laos) ports in 2014, respectively [9]. The large moving population caused dengue outbreak in Ruili in 2008 and Mengla in 2013 [20, 21]. In addition, due to the armed conflict in Myanmar, events in which a large number of refugees crossed the border into the Ruili area occurred several times, which increased the threat to the local disease prevention system. Thus, it is urgent to enhance the capabilities of local health institutes for infectious disease surveillance and outbreak response. Furthermore, in recent years, the number of female migrant workers have continued to increase and have exceeded male workers in the area [9], which has caused female cases to increase rapidly in recent years. In addition, marriage migration is also a significant trend in terms of Burmese migration into China. As estimated, there were more than 25 000 Burmese brides living in Yunnan [22]. This phenomenon may frequently cause cross-border travel among family members, which affects all age groups within the family. Thus, specific intervention measures should be carried out to focus on the high-risk groups of female migrant workers and families with cross-border marriages.

The main limitation of this study was that the annual entry-exit population for each county was not obtained, which affected the assessment of the incidence rates of the imported diseases. In addition, the fragmentary epidemiological information for each case limited further analysis, such as the identification of risk factors for infection. Thus, detailed investigations should be designed for future intervention improvements.

## Conclusion

In conclusion, different epidemiological aspects of acute infectious diseases were explored between foreign and native imported cases in the border counties. The disease spectrum was more diverse among foreign cases than among native cases. Both foreign and native cases were mostly of Myanmar origin and native farmers and businessmen, foreign women and preschool children were frequently detected with infections. Thus, stronger disease screening at the border is urgently necessary to reduce the risk of cross-border disease transmission, particularly at the Myanmar border. Health education interventions should focus on native farmers and businessmen to avoid possible exposures when they travel abroad. In addition, healthcare services should be provided to foreign migrant women and preschool children to reduce possible autochthonous spreading. Moreover, international collaboration in terms of sharing related infectious disease information, providing professional training and coordinating outbreak control responses should be improved in the region.

## Supplementary material

For supplementary material accompanying this paper visit https://doi.org/10.1017/S0950268819001195. click here to view supplementary material

## Availability of data and materials

The datasets used and analysed during the current study available from the corresponding author on reasonable request.

## References

[1] China National Tourism Administration. Big data on the overseas travelling in China in 2016. Available at http://www.cnta.gov.cn/xxfb/hydt/201701/t20170124_813152.shtml (Accessed 29 July 2018).

[2] Mirski T, Bartoszcze M and Bielawska-Drozd A (2011) [Globalization and infectious diseases]. Przeglad Epidemiologiczny 65, 649–655, (in Polish). 22390054

[3] Wang YL (2017) [Epidemiology of imported infectious diseases in China, 2013–2016]. Zhonghua liu xing bing xue za zhi 38, 1499–1503, (in Chinese). 29141337 10.3760/cma.j.issn.0254-6450.2017.11.012

[4] Statistics Bureau of China (2017) China Statistical Yearbook *(2016)*. Beijing, China: China Statistics Press.

[5] Ahn C and Chang J (2017) Cross-border trade and marriage : case study of a Chinese Yunnan Minority Village. Journal of Chung-Ang Historical Studies 46, 625–655.

[6] Xinhuanet. Yunnan border defence. Available at http://www.yn.xinhuanet.com/newscenter/2018-02/11/c_136966189.htm (Accessed 4 August 2018).

[7] Phommasack B (2013) Mekong basin Disease Surveillance (MBDS): a trust-based network. Emerging Health Threats Journal 6, 19944. 10.3402/ehtj.v6i0.19944 PMC3557908 23362411

[8] Fang LQ (2018) Travel-related infections in mainland China, 2014-16: an active surveillance study. The Lancet Public Health 3, e385–e394. 30033200 10.1016/S2468-2667(18)30127-0 PMC7164813

[9] Liu Y, Hu Z and Ge Y (2017) [Spatial difference and contributing factors of the foreign workers of border ports in Yunnan]. Tropical Geography 37, 174–184, (in Chinese).

[10] You W and Zhang X (2018) [Management problems in the borderland of Yunnan concerning the cross-border education of the school children from China's neighboring countries]. Journal of Yunnan Normal University (Humanities and Social Sciences) 3, 102–109, (in Chinese).

[11] Zhi Z and Shen P (2018) [The study on cross-border people movement at Yunnan border area]. Xue Shu Tan Suo 5, 69–75, (in Chinese).

[12] Wang X (2016) Effects of a malaria elimination program: a retrospective study of 623 cases from 2008 to 2013 in a Chinese county hospital near the China--Myanmar border. Emerging Microbes & Infections 5, e6. 26785944 10.1038/emi.2016.6 PMC4735059

[13] Mekong Migration Network (MMN) and Asian Migrant Centre (AMC). Migration in the greater mekong subregion. Available at http://www.mekongmigration.org (Accessed 25 August 2018).

[14] World Health Organization. Mekong Malaria Elimination (MME) programme. Available at http://www.who.int/malaria/areas/greater_mekong/en/ (Accessed 25 August 2018).

[15] Xinhuanet. No malaria cases caused by mosquito bites in China in 2017. Available at http://english.cqnews.net/html/2018-04/25/content_44202395.htm (Accessed 26 August 2018).

[16] Bhatt S (2013) The global distribution and burden of dengue. Nature 496, 504–507. 23563266 10.1038/nature12060 PMC3651993

[17] Ooi EE and Gubler DJ (2009) Dengue in Southeast Asia: epidemiological characteristics and strategic challenges in disease prevention. Cadernos de saude publica 25(suppl. 1), S115–S124. 19287856 10.1590/s0102-311x2009001300011

[18] Kuan MM (2010) Epidemiological trends and the effect of airport fever screening on prevention of domestic dengue fever outbreaks in Taiwan, 1998–2007. International Journal of Infectious Diseases 14, e693–e697. 20656647 10.1016/j.ijid.2009.12.010 PMC7110484

[19] Zaw PPT (2015) Disparities in health and health care in Myanmar. Lancet 386, 2053. 26700385 10.1016/S0140-6736(15)00987-3 PMC4672190

[20] Zhang HL (2013) [An outbreak of imported dengue fever from Myanmar to the border of China, with its viral molecular epidemiological features]. Zhonghua liu xing bing xue za zhi 34, 428–432, (in Chinese). 24016428

[21] Wang B (2016) The distinct distribution and phylogenetic characteristics of dengue virus serotypes/genotypes during the 2013 outbreak in Yunnan, China: phylogenetic characteristics of 2013 dengue outbreak in Yunnan, China. Journal of Molecular Epidemiology and Evolutionary Genetics in Infectious Diseases 37, 1–7. 26597450 10.1016/j.meegid.2015.10.022

[22] Hackney L (2015) Re-evaluating Palermo: the case of Burmese women as Chinese brides. Anti-Trafficking Review 4, 98–119.

