# Supplementary material for: Comparison of the epidemiological aspects of acute infectious diseases between foreign and native imported cases in the border counties of Southwest China, 2008–2017
Source: Epidemiol Infect. 2019 Jun 28;147:e230. doi: 10.1017/S0950268819001195 (PMC6625214; doi:10.1017/S0950268819001195)
Supplement: Supplementary file 1 [file S0950268819001195sup001.docx]

| id | disease | latent period | reference |
| --- | --- | --- | --- |
| 1 | malaria | 11 days - 1 year | WS 259-2015 |
| 2 | dengue fever | 1 - 14 days | WS 216-2018 |
| 3 | Japanese encephalitis | 4 - 21 days | WS 214-2008 |
| 4 | scrub typhus | 4 - 14 days | WS 215-2008 |
| 5 | hand-foot-and-mouth disease | 2 - 10 days | WS 588-2018 |
| 6 | viral hepatitis (A or E) | 14 - 49 days | WS 298-2008 |
| 7 | shigellosis | few hours - 7 days | WS 287-2008 |
| 8 | other infectious diarrheas (rule out cholera, typhoid and paratyphoid, shigellosis) | few hours - 14 days | WS 271-2007 |
| 9 | typhoid and paratyphoid | 1 - 21 days | WS 280-2008 |
| 10 | cholera | 1 - 7 days | WS 289-2008 |
| 11 | influenza | 1 - 7 days | WS 285-2008 |
| 12 | measles | 7 - 21 days | WS 296-2017 |
| 13 | mumps | 14 - 28 days | WS 270-2007 |
| 14 | rubella | 14 - 21 days | WS 290-2008 |
| 15 | scarlatina | 2 - 5 days | WS 282-2008 |
| 16 | pertussis | 2 - 21 days | WS 274-2007 |
| 17 | epidemic cerebrospinal meningitis | few hours - 10 days | WS 295-2008 |
| 18 | gonorrhea | 1 - 10 days | WS 268-2007 |
| 19 | acute hemorrhagic conjunctivitis | few hours - 2 days | WS 217-2008 |
| 20 | rabies | 10 days - 1 year | WS 281-2008 |
| 21 | tetanus | 3 -14 days | WS 272-2007 |
| 22 | leptospirosis | 1 - 30 days | WS 290-2008 |
| 23 | cutaneous anthrax | 1 - 3 days | WS 283-2008 |
|  | WS: Guideline of diagnosis issued by health ministry of China | | |
